# Homogeneous Polyporus Polysaccharide Inhibit Bladder Cancer by Resetting Tumor-Associated Macrophages Toward M1 Through NF-κB/NLRP3 Signaling

**DOI:** 10.3389/fimmu.2022.839460

**Published:** 2022-05-04

**Authors:** Chunping Liu, Dongyue He, Shihui Zhang, Huiqi Chen, Jie Zhao, Xiong Li, Xing Zeng

**Affiliations:** ^1^ State Key Laboratory of Dampness Syndrome of Chinese Medicine, The Second Affiliated Hospital of Guangzhou University of Chinese Medicine, Guangzhou, China; ^2^ Guangdong-Hong Kong-Macau Joint Lab on Chinese Medicine and Immune Disease Research, Guangzhou, China; ^3^ State Key Laboratory of Quality Research in Chinese Medicine, Institute of Chinese Medical Sciences, University of Macau, Macau, China; ^4^ Artemisinin Research Center, Guangzhou University of Chinese Medicine, Guangzhou, China

**Keywords:** homogeneous polyporus polysaccharide, bladder cancer, M1 subtype, tumor-associated macrophages, NF-κB/NLRP3 signaling

## Abstract

Bladder cancer(BC)is one of the most common urinary system tumors, which characterized by a high incidence. Polyporus polysaccharide is the main active component of polyporus, which is clinically used in the treatment of bladder cancer, but the mechanism is not clear. In previous study, we isolated homogeneous polyporus polysaccharide(HPP) with high purity from polyporus. The goal of this study was to assess the polarization of macrophages induced by HPP in the bladder tumor microenvironment and explored its anti-bladder cancer mechanism through BBN bladder cancer rat model and Tumor associated macrophages(TAM). The results suggested that HPP regulates TAM polarization to improve the tumor inflammatory microenvironment, possibly through the NF-κB/NLRP3 signaling pathway. Our results suggested that HPP may be a potential therapeutic agent for bladder tumors.

## Background

Bladder cancer(BC) is one of the most common urinary system tumors, ranking fourth in the incidence of male urinary system tumors (1). It is characterized by a high incidence, recurrence and mortality rate, and its treatment is mainly based on surgery combined with radiotherapy and chemotherapy. BCG is one of the most effective biological agents in the treatment of bladder cancer and is the first choice for bladder infusion (2). Clinical studies found that the recurrence rate of bladder cancer after BCG infusion was lower than that of mitomycin C (3). However, while the curative effect of BCG is good, it is accompanied by serious side effects and 30-50% of patients would relapse within 5 years (1, 4, 5). Therefore, identification of new bladder infusion drug with high efficiency and low toxicity for BC treatment is urgently needed.

The etiology and pathogenesis of BC are complex and have not been fully elucidated at present. Increasing reports suggest that the Tumor microenvironment (TME) plays a vital role in the occurrence and deterioration of BC. TEM is composed of tumor cells, stromal cells, immune cells and various biological factors, and macrophages are one of the most important regulators. Macrophages are a homogenous population whose phenotypes are determined by biological factors in the microenvironment. Macrophages can be divided into classical M1 and non-classical M2 subtypes according to phenotypes and secreted cytokines (6). M1 subtype Macrophages secrete pro-inflammatory factors to promote immune response and enhance the effect of killing tumors (7, 8), while M2 lead to anti-inflammatory effects and provide conditions for tumor immune escape (9, 10). Studies found that macrophages can be polarized to the M1 subtype by STATE1, NF-κB, and NLRP3 signaling (8, 11). Tumor-associated macrophages(TAMs) infiltrate tumor tissues and are widely distributed in the tumor microenvironment (12), which is similar to the M2 subtype, can promote tumor growth, invasion and angiogenesis. The relationship between dysfunctions of macrophages and bladder cancer play a key role to be solved in immunotherapy (13).

Polyporus, a medicinal fungus, the main function is diuretic in clinic (14). In addition, it also has a variety of pharmacological effects, such as enhancing immune function, antitumor activity, inhibiting mesangial growth, anti-urinary tract infection and anti-inflammation (15). Polyporus polysaccharide (PPS) is one of the main active components in polyporus (14). In the 1970s, PPS was isolated by Japanese scholars for the first time (16). Subsequent studies demonstrated that PPS could inhibit transplanted tumors in animals. In recent years, it has been found that PPS can improve human immunity and is widely used as an immune-potentiator (9, 15). PPS injection regulates immune function and is used in the treatment of bladder cancer, lung cancer and blood cancer, etc. Combined with anti-tumor chemotherapeutic drugs, it can obviously enhance curative effect and reduce toxic and side effects. Although polyporus polysaccharide injection has good efficacy, some tumor patients have some adverse reactions such as joint damage, which limits its use. It was closely related to the fact that PPS was a mixture and the active substance base was unclear. Polyporus polysaccharide injection is a mixture, and the unclear substance basis is an important reason for its adverse reaction. In our previous study, homogeneous polyporus polysaccharide (HPP) was first isolated from polyporus total polysaccharide, which was proven to have an α-(1→ 4)-linked D-galactan backbone. Our previous experimental results found that HPP -treated macrophage can promote the apoptosis and necrosis of bladder tumor cells (10). In this study, we explored the mechanism by which HPP regulates macrophages to inhibit bladder cancer. In a previous study, we found that PPS could enhanced the expression of TLR2 and greatly induce the transformation of M2 subtype macrophages to the M1 subtype *in vitro* (17). The classic pathway of polysaccharides activation of macrophages is NF-κB, and NLRP3 is one of the downstream pathways of NF-κB. Our study found that HPP may promote the polarization of macrophages by regulating the TLR2/NF-κB/NLRP3 signaling pathway, inhibiting the immune-suppression of TME.

The purpose of this study was to explore the antitumor effect of HPP on bladder cancer and the possible mechanism by which HPP promotes the polarization of macrophages toward M1 in the tumor microenvironment. The results of this study may provide new immune strategies for the development and utilization of polysaccharides in the treatment of bladder cancer.

## Materials and Methods

The mouse macrophage cell lines RAW 264.7, human monocytic leukemia THP-1, and transitional cell carcinoma of the bladder cell line T24 were purchased from ATCC (Rockville, MD, USA). Specific monoclonal antibodies, including antibodies against iNOS, NLRP3, IκB-α, p-IκB, p-Iκκ-α/β, Cox2, p65, p-P65 and GAPDH, were purchased from Cell Signaling Technology Inc. (Beverly, MA, USA). Secondary antibodies for western blotting and 3-(4,5-dimethylthiazol-2-yl)-2,5-diphenyltetrazolium bromide (MTT) were purchased from Sigma (Sigma Aldrich, St. Louis, USA). Fetal bovine serum (FBS), Dulbecco’s modified Eagle’s medium (DMEM) and penicillin/streptomycin solution were purchased from HyClone (Logan, UT, USA). Phycoerythrin (PE)-conjugated anti-CD40, CD16/32, fluorescein isothiocyanate (FITC)-conjugated anti-mouse CD282 and FITC-IgG2a antibodies were purchased from BD Systems (BD Biosciences, USA). ProcartaPlex**™** Multiplex Immunoassay Kits were purchased from eBioscience (San Diego, CA, USA). DEAE-52 and Sephadex G-100 gel filtration medium was purchased from GE Health Care BioSciences AB (UPPPala, Sweden). SC75741 (purity, 99.79%) was purchased from Selleck (Shanghai, China).

### Animal Model

Fischer-334 rats were purchased from Vital River Laboratory Animal Technology Co., Ltd. (Beijing, China). The most classical way of bladder cancer administration is bladder perfusion. At present, bladder perfusion in mice is not convenient, and the way of intravenously inserted needle retention in mice is easy to cause inflammatory reaction, which is not conducive to the study of immune response caused by polysaccharides. Fischer-334 rats have similar genetic background and are one of the most classical animal models of bladder cancer. In our previous experiments, BBN bladder cancer rat model have been used for many times. Preparation of the animal model (18): OH-BBN was dissolved in 20:80 (ethanol:water). Except for the blank group, animals administering models at 150 mg/time twice a week and the body weight was weighed every day. After the modeling was completed, the water extracts of polyporus and polysaccharide were given by gavage (19). Animal grouping and treatment: The rats were immediately divided into 4 groups, namely, the Control group, the model group, the water decoction of polyporus (WDP) group, and the polyporus polysaccharide group (PPS). Observation index: Rat bladder tissue was collected and stored or embedded at -80°C. The rat bladder was stained for routine pathological section observation, and immune-histochemical staining was performed to observe the expression of related proteins in the tumor microenvironment.

### Histopathology

Histological analysis was used to observe the change in pathological conditions in the bladder tissue of rats. Paraffin-embedded bladder sections 4 µm thick were HE stained, and then, a blinded pathologist analyzed them. On the basis of the Geboes criteria, the severity of UC was graded from 0 to 5. Histopathological results were determined on the basis of the extent of inflammation, ulceration and epithelial damage as described previously.

### Immunohistochemistry (IHC)

Immuno-histochemical assays were used to determine CD163 and iNOS protein expression in the bladder tissue of rats. Tissues were obtained from rats with/without HPP treatment, fixed in 10% formaldehyde for 24 h, and then embedded in paraffin. The specimens were cut into 5-μm sections and roasted at 60°C for 2 h. Antigenic retrieval was performed in citric acid buffer (pH = 6.0) followed by washing with PBS and treatment with 3% H_2_O_2_ and 5% bovine serum albumin. Afterward, the sections were incubated with primary antibodies against CD163 and iNOS (dilutions of 1:50; Cell Signaling Technology) at 4°C overnight. Subsequently, they were incubated with secondary antibody for 30 min. Detection was performed using 3,3′-diaminobenzidine (DAB) chromogen (Maixin Biotech. Co. Ltd, Fuzhou, China), and the sections were counterstained with hematoxylin, dehydrated through graded alcohols, and cleared in xylene. Finally, pictures were taken under 200× magnification under a microscope (Ti2-E; Nikon). Immunostaining was evaluated by Image-Pro Plus 6.0 image analysis software (Media Cybernetics, Inc., Silver Spring, MD, USA).

### Cell Line Culture and Tumor-Conditioned Media Preparation

RAW 264.7, TPH-1 and T24 cells were cultured in DMEM with 10% FBS containing 1% penicillin/streptomycin. For all experiments, cell lines were cultured at 37°C in a humidified atmosphere containing 5% CO2. The T24 supernatant was collected and filtered at 0.22 µm, after which the supernatant was stored at -80°C. Once the cells reached 80% confluence, 40% of the medium was discarded, and the cells were incubated with fresh DMEM or T24 supernatant for 3 h. Finally, the samples were treated with or without different concentrations of HPP for 24 h, with equal volumes of medium used as controls.

### Isolation and Purification of Polysaccharides

The new polysaccharide was first isolated from polyporus total polysaccharide using Sephadex G-100 gel-filtration column (2.9 × 50cm), which was proven to have an α-(1→ 4)-linked D-galactan backbone (20).

### Measurement of Macrophage-Derived Cytokine Production

RAW 264.7 cells or THP-1 cells were seeded in 12-well plates and cultured for 24 h. The cells were then incubated with 50% fresh medium or T24 cell culture supernatant for 3 h before treatment with HPP for 24 h. After the treatments, the culture medium was replaced with fresh medium, the cells were cultured for an additional 48 h, and then the concentrations of NO, IL-6 and TNF-α in the culture medium were evaluated. Nitrite accumulation in the culture supernatant was measured based on the Griess reaction. In addition, the levels of IL-6, TNF-α, IL-23, MIP-1α, MIP-1β and IL-1β in the culture supernatant were assayed using a CBA assay kit according to the manufacturer’s instructions.

### Flow Cytometry Analysis

Flow cytometry was performed to determine macrophage phenotypes. In brief, macrophages were plated in 12-well culture plates for 24 h, and afterward, the culture medium was replaced with T24 cell culture supernatant for 3 h before incubation with different concentrations of HPP (1, 10 and 100 µg/mL) for 24 h. Next, we harvested the treated cells and washed them twice with cold phosphate-buffered saline (PBS). Then, 5 µL of CD40, CD16/32, and FITC-conjugated anti-mouse CD282, PE-IgG2a and FITC-IgG2a antibodies were added to the tubes and incubated for 20 min on ice. Finally, the stained cells were suspended in cold buffer, and fluorescence-activated cell sorting (FACS) was used to analyze the data.

### Quantitative Real-Time Polymerase Chain Reaction (qRT–PCR)

qRT–PCR assays were conducted to examine the mRNA expression of iNOS, IL-1β, TNF-α, IL-6 and GAPDH. Cells were washed three times in cold PBS before being harvested with TRIzol reagent (Ambion, MA, USA). A NanoDrop Lite spectrophotometer (Thermo Scientific, MA, USA) was used to detect the concentration and purity of the total RNA. For the detection of NOS, IL-1β, TNF-α, IL-6 and GAPDH mRNA expression, reverse transcription was performed using a Transcriptor First Strand cDNA Synthesis Kit (Roche, Basel, Switzerland), and PCR was performed using FastStart Universal SYBR Green Master Mix (ROX) (Roche, Basel, Switzerland) according to the manufacturer’s protocols. All primers used in this study were designed as listed in **Supplementary Material 1**.

### Western Blot Analysis

Western blot assays were used to analyze protein expression. After treatment with the indicated HPP (1, 10 and 100 µg/mL), cells were harvested and lysed with 1× RIPA buffer (CST. Beverly, USA) with Complete Protease Inhibitor Cocktail (Roche, Switzerland). The protein concentrations were measured using the Pierce BCA Protein Assay Kit (Thermo Fisher Scientific, Inc.). Equal amounts of protein from whole cell lysates were solubilized in 3×loading buffer (CST, USA), separated by 10% SDS-PAGE, and then transferred onto polyvinylidene difluoride (PVDF) membranes (Millipore, Burlington, MA, USA). The PVDF membranes were incubated with antibodies against iNOS, NLRP3, IκB-α, p-IκB, p-Iκκ-α/β, Cox2, p65, p-P65, and GAPDH at 4°C overnight. Subsequently, the membranes were washed and incubated with a secondary antibody for 1 h at room temperature. The membranes were washed again and transferred to a freshly prepared enhanced chemi-luminescence solution (Millipore, MA, USA). Signals were then observed and scanned under the Bio-Rad Chemi-Doc Touch Chemi-luminescence imaging system (Bio-Rad Laboratories, Inc.). The results were analyzed using ImageJ software (version 1.48; National Institutes of Health).

### Inhibition of NF-κB Using a Specific Inhibitor

To identify the signal transduction pathways that mediate the effects of PPS on IFN-γ-stimulated macrophage activation, RAW 264.7 cells were pretreated with the NF-κB inhibitor SC75741 (5 μM) for 2 h in DMEM or a co-culture microenvironment. This medium was subsequently replaced with medium with or without IFN-γ (100 ng/ml) for 3 h. Then, the cells were treated with PPS (250 μg/mL) for 8 h. After treatment, the cells were harvested and analyzed by western blot.

### Statistical Analysis

All data are expressed as the mean ± standard error of the mean (S.E.M.) of three independent experiments. Statistical analysis was performed using Student’s *t*-test when there were only two groups (two-sided). Differences between groups were assessed by one-way ANOVA followed by Tukey’s multiple comparison test for multiple groups involved (Graph-pad Prism 7.0 software, CA, USA). *P* values < 0.05 were considered statistically significant.

## Results

### Therapeutic Effect of Polyporus on BBN Bladder Cancer Rats

**Figure 1:**
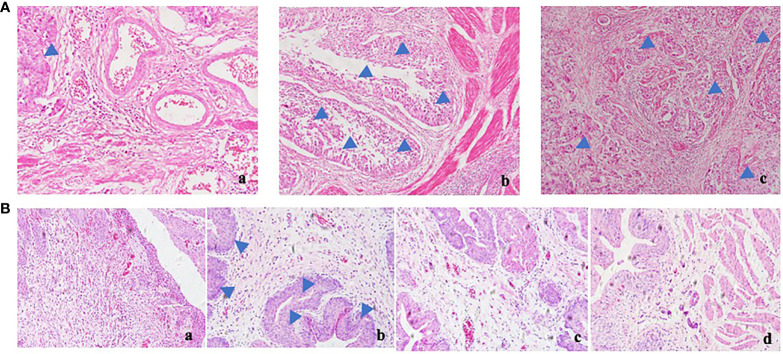
Effect of polyporus on BBN bladder cancer rats. **(A)** HE staining of human bladder tumor specimens. a. paracancerous b, c. tumor tissue. **(B) **HE staining of BBN rats after intervention with polyporus extract and polysaccharide. a. Control group b. Model group c. PPS group d. WDP group.

BBN rats were given polyporus water extract and polysaccharide intervention, and the control group and model group were given normal saline intergalactic administration. The weight of the rats was recorded every day. As shown in **Figure 1B**, the body weight of BBN bladder cancer rats showed a decreasing trend, while the BBN rats in the polyporus water extract and polysaccharide groups showed an increasing trend, which was similar to the control group, suggesting that polyporus intervention can improve the quality of life of bladder cancer rats. Histological changes in the bladder in BBN bladder cancer rats were observed by HE staining. As shown in **Figure 1B**, the bladder wall of rats in the control group was composed of three layers of tissue, including the mucosal layer, muscle layer and outer membrane from inside to outside, and the muscle layer was composed of smooth muscle fibers. In the model group, the cancer cells penetrated through the basement membrane and entered the submucosa, infiltrating into the muscle layer, the fat outside the bladder and even the peritoneum, and the smooth muscle tissue was squeezed and deformed, which was similar to the pathological results of the bladder tumor shown in **Figure 1A**, indicating that our bladder cancer model was successfully constructed. The pathological changes in HE sections of BBN rats were significantly improved after intervention with polyporus extract and polysaccharide, suggesting that polyporus extract and PPS only have good therapeutic effects on bladder tumors.

### Effect of Polyporus on the Tumor Microenvironment and Establishment of the TAM Model *In Vitro*

**Figure 2:**
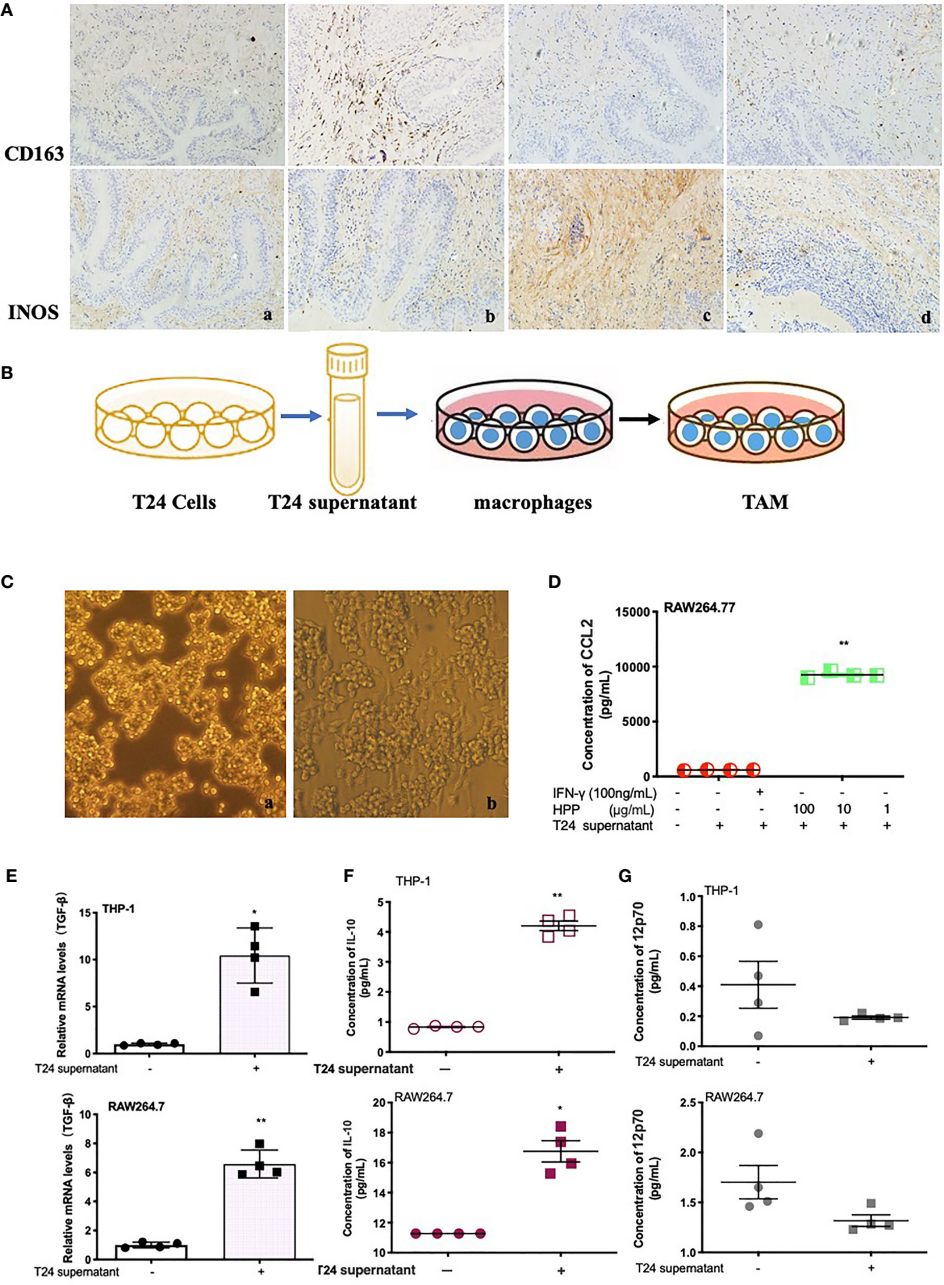
Effect of polyporus on the tumor microenvironment and establishment of the TAM model *in vitro*. **(A)** Immunostaining of CD163 and iNOS. a. Control group, b. Model group, c. PPS group, d. WDP group. **(B)** Schematic diagram of macrophage co-culture with T24 supernatant. **(C)** Microscopy images of IFN-γ-stimulated RAW 264.7 cells in medium or a co-culture microenvironment. a. RAW264.7 group, b. RAW264.7+T24 supernatant group. **(D)** CCL2 secretion in stimulated RAW 264.7 cells treated with T24 supernatant. **(E)** The expression of TGF-β mRNA in macrophages treated with T24 supernatant, followed by measuring the mRNA levels by qRT–PCR. **(F, G)** The secretion of IL-10 and 12p70 in macrophages treated with T24 supernatant. Values are given as the mean ± SD from three independent experiments. Compared with the control group, *P* < 0.05, ^**^ *P* < 0.01.

Tissue sections were stained with IHC, as shown in **Figure 2A**. Compared with the model group, the polyporus water extract and polysaccharide intervention group downregulated CD163 protein and upregulated iNOS protein expression in bladder tissues of BBN bladder cancer rats, suggesting that polyporus could regulate the transformation of TAMs to the M1 subtype. To further explore the regulatory mechanism of TAMs by polyporus, we co-cultured bladder cancer cell supernatant and macrophages to simulate macrophages in the tumor microenvironment, as shown in **Figure 2B**. In addition, a co-culture model of THP-1 and T24 supernatant was established in our previous experiments (21). As shown in **Figure 2C**, RAW264.7 macrophages were round or oval under the microscope when they were not stimulated, with clear cell boundaries, good adherence and few protrusions. After T24 supernatant intervention, the volume of macrophages increased significantly, and some vacuoles appeared. Increased secretion of IL-10 and CCL2 and low secretion of IL-12p70 are typical characteristics of M2 like macrophages. The cytokine secretion of the co-culture model was detected by the liquid chip method. Tumor supernatants of bladder cancer cells promoted the secretion of IL-10 and CCL2 but had no effect on IL-12p70 (**Figures 2D, F, G**). TGF-β is another important indicator of the M2 subtype. Tumor supernatant intervention in bladder carcinoma cells promoted TGF-β mRNA expression in macrophages, as shown in **Figure 2E**.

### Polyporus and Polyporus Polysaccharides Drives TAMs Toward the M1 Subtype

**Figure 3:**
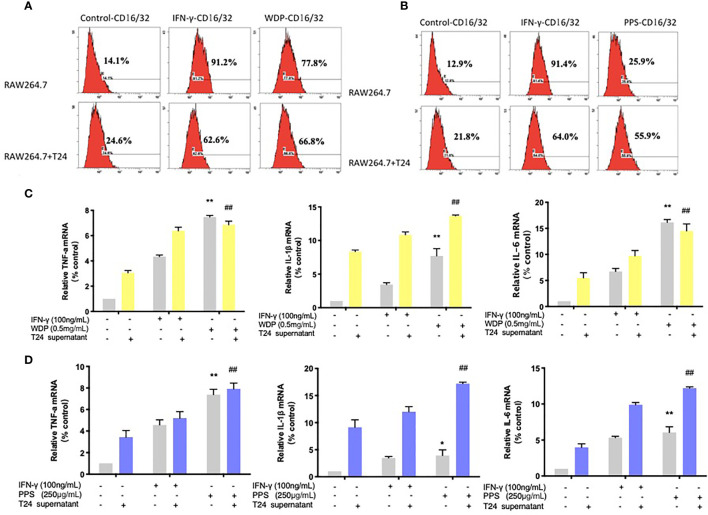
Polyporus water extract and polysaccharides promoted TAMs toward the M1 subtype. **(A, B)** Flow cytometry detection of CD16/32, the signature marker of M1 cells. **(C)** The expression of IL-1β, TNF-α, and IL-6 mRNA in RAW 264.7 cells treated with polyporus WDP for 24 h, followed by measuring the mRNA levels by qRT–PCR. **(D)** The expression of IL-1β, TNF-α, and IL-6 mRNA in RAW 264.7 cells treated with PPS for 24 h, followed by measuring the mRNA levels by qRT-PCR. Values are given as the mean ± SD from three independent experiments. Compared with the RAW 264.7 control group, *^##^P* < 0.01; compared with the RAW 264.7+T24 control group, *P < 0.05 and **P < 0.01.

Membrane phenotype molecules are important indicators of macrophage polarization, and CD16/32 and CD40 are membrane molecules of the M1 macrophage subtype. As shown in **Figures 3A, B**, flow cytometry results showed that PPS and WDP increased the expression of TAM CD16/32 membrane molecules in a concentration-dependent manner. The positive expression rates of CD16/32 membrane surface molecules in the PPS group were 40.2%, 55.3%, and 64.2%. The positive expression rates of CD16/32 surface molecules in the polyporus water extract group were 33.3%, 62.1% and 67.6%, respectively. Il-1β, TNF-α and IL-6 mRNA are the classical pro-inflammatory molecules of M1 macrophages. As shown in **Figures 3C, D**, the mRNA expression of TAM IL-6, TNF-α and IL-1β in the PPS and WDP intervention groups was significantly upregulated compared with that in the control group (*P <*0.05).

The results suggest that PPS and WDP can promote the transformation of TAM cells into M1 macrophages in the tumor microenvironment, but the effective substances are not clear.

### Configuration of HPP and the Activation of Macrophages

**Figure 4:**
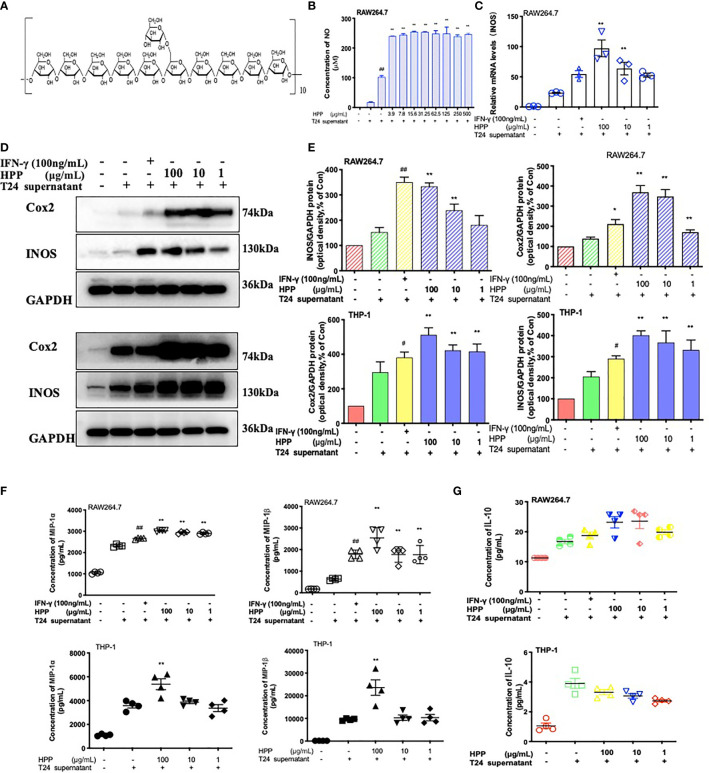
Configuration of HPP and the activation of macrophages. **(A)** Configuration of HPP. **(B)** NO secretion in macrophages treated with HPP. **(C)** The expression of iNOS mRNA in macrophages stimulated by HPP. **(D, E)** The expression of iNOS and Cox2 in macrophages stimulated by HPP. **(F)** The secretion of MIP-1α and MIP-1β in macrophages treated with HPP. **(G)** The secretion of IL-10 in macrophages treated with HPP. Values are given as the mean ± SD from three independent experiments. Compared with the control group, ^#^ *P* < 0.05; compared with the RAW 264.7+T24 group, ^*^ *P* < 0.05.

HPP was extracted by the glucan gel method and was composed of monosaccharides (glucose), as shown in **Figure 4A**. Its configuration was all α type, and the main connection mode was α 1→4 connection. In the tumor microenvironment, macrophages were co-cultured with different concentrations of HPP for 24 h, the culture supernatant was replaced with complete medium, and the cell supernatant was collected for 24 h. The content of NO in the supernatant was determined by the Griess method. As shown in **Figure 4B**, NO secretion increased in a concentration-dependent manner in the HPP intervention group. In addition, PCR results showed that HPP increased the expression of TAM iNOS mRNA (**Figure 4C**). Meanwhile, WB results showed that HPP presented a dose-dependent upregulation of iNOS and Cox2 protein expression in the tumor microenvironment (**Figures 4D, E**). Macrophage inflammatory proteins MIP-1α and MIP-1β are markers of macrophage activation, and it was found that HPP presented dose-dependent upregulation of TAM IL-10, MIP-1α and MIP-1β (**Figures 4F, G**). Based on the above experimental results, we found that HPP could induce the activation of macrophages in the tumor microenvironment, possibly promoting the polarization of M1 macrophages.

### HPP Drives TAMs Toward M1 Subtypes

**Figure 5:**
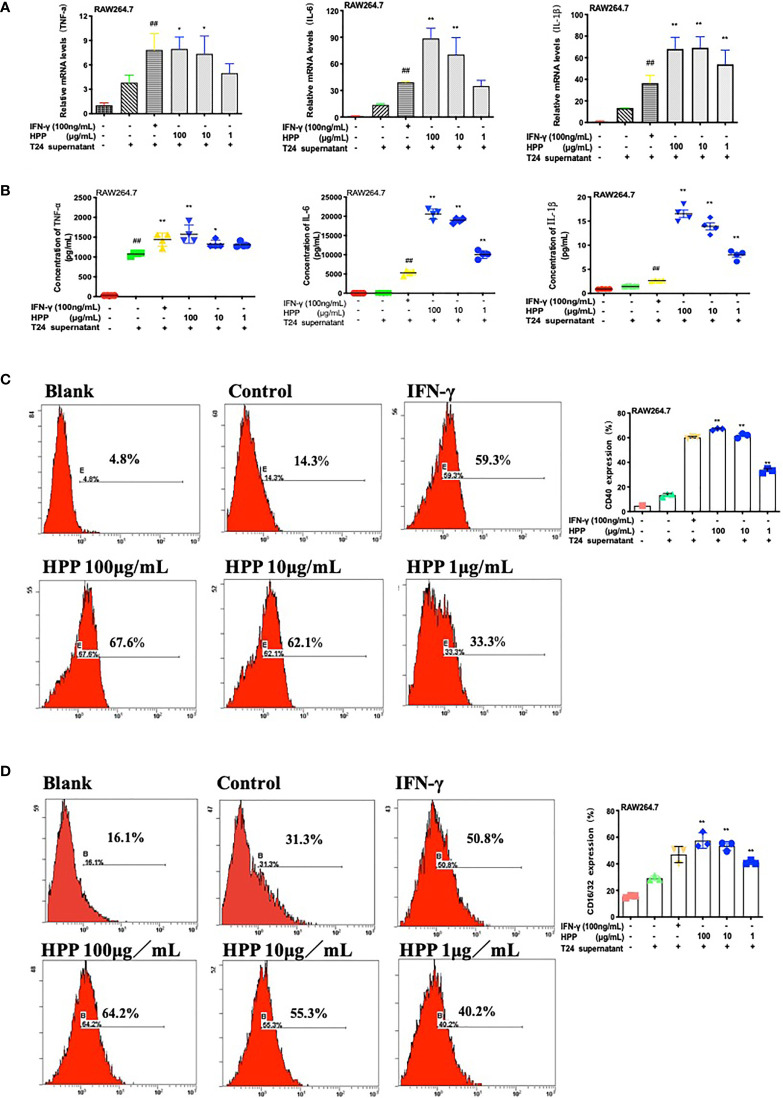
HPP drives TAMs toward the M1 subtype. **(A)** The expression of IL-1β, TNF-α, and IL-6 mRNA in RAW 264.7 cells treated with HPP for 24 h, followed by measuring the mRNA levels by qRT–PCR. **(B)** The secretion of IL-1β, TNF-α, and IL-6 in macrophages treated with HPP. **(C, D)** Flow cytometry detection of CD40 and CD16/32, the signature markers of M1 cells. Values are given as the mean ± SD from three independent experiments. Compared with the control group, ^##^ *P* < 0.01; compared with the RAW 264.7+T24 control group, *P < 0.05 and **P < 0.01.

Macrophages of the M1 subtype are characterized by high expression of IL-1β, IL-6 and TNF-α mRNA. A real-time PCR experiment was conducted. As shown in **Figure 5A**, PCR results showed that HPP promoted the expression of IL-1β, IL-6 and TNF-α mRNA in macrophages in the tumor microenvironment. Further liquid chip experiments, as shown in **Figure 5B**, showed that HPP promoted the secretion of inflammatory factors such as TAMs IL-6, IL-1β and TNF-α. CD16/32 and CD40 are markers of macrophage polarization. Phenotypes of macrophages were identified. As shown in **Figures 5C, D**, flow cytometry results showed that HPP promoted the polarization of TAMs toward M1. Based on the above experimental results, we found that HPP could promote the polarization of macrophages toward the M1 subtype in the tumor microenvironment.

### NF-κB/NLRP3 Pathway Involvement in Macrophage Polarization Induced by HPP

**Figure 6:**
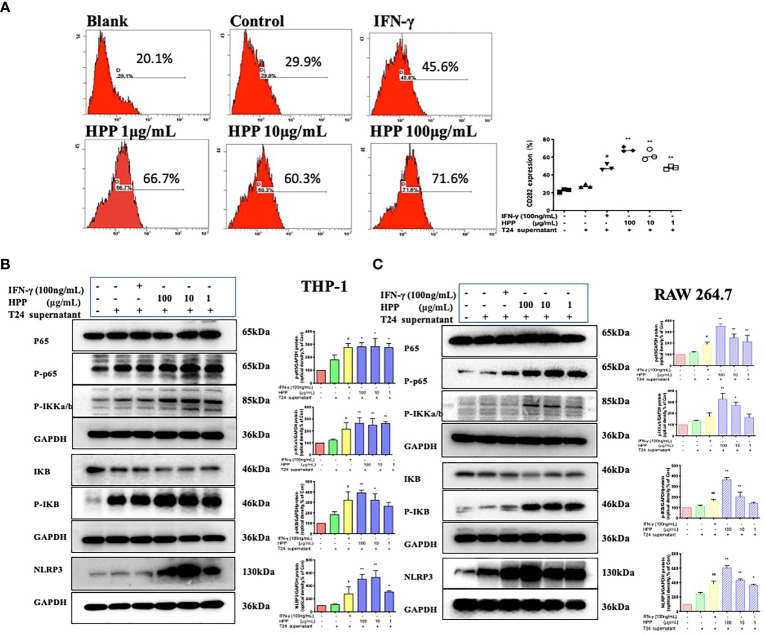
Effect of HPP on the NF-κB/NLRP3 pathway in macrophages. **(A)** The expression of CD282 (TLR2) in macrophages treated with HPP for 24 h. Cells were collected and processed for the analysis of the cell cycle distribution by flow cytometry. **(B)** Effect of HPP on NF-κB/NLRP3 pathway in THP-1 macrophages. And p-IκB, p-IKK-α/β, p-P65 and NLRP3 were determined by western blot. **(C)** Effect of HPP on NF-κB/NLRP3 pathway in RAW 264.7 macrophages. And p-IκB, p-IKK-α/β, p-P65 and NLRP3 were determined using western blotting. Values are given as the mean ± SD from three independent experiments. Compared with the control group, ^#^ *P* < 0.05, and ^##^ *P* < 0.01; compared with the RAW 264.7+T24 control group, *P < 0.05 and **P < 0.01.

In the tumor microenvironment, HPP upregulated the expression of the TLR2 receptor in macrophages in a dose-dependent manner. Further studies showed that HPP activated the NF-κB/NLRP3 pathways downstream of TLR2 and upregulated the protein expression of p-IκB, p-IKK-α/β, p-P65 and NLRP3. As shown in **Figure 6**, the polarization of TAMs toward M1 by HPP in the tumor microenvironment may be mediated by the NF-κB/NLRP3 pathway.

### Inhibition of the NF-κB Pathway Reverses the Activation of Macrophages Induced by HPP

**Figure 7:**
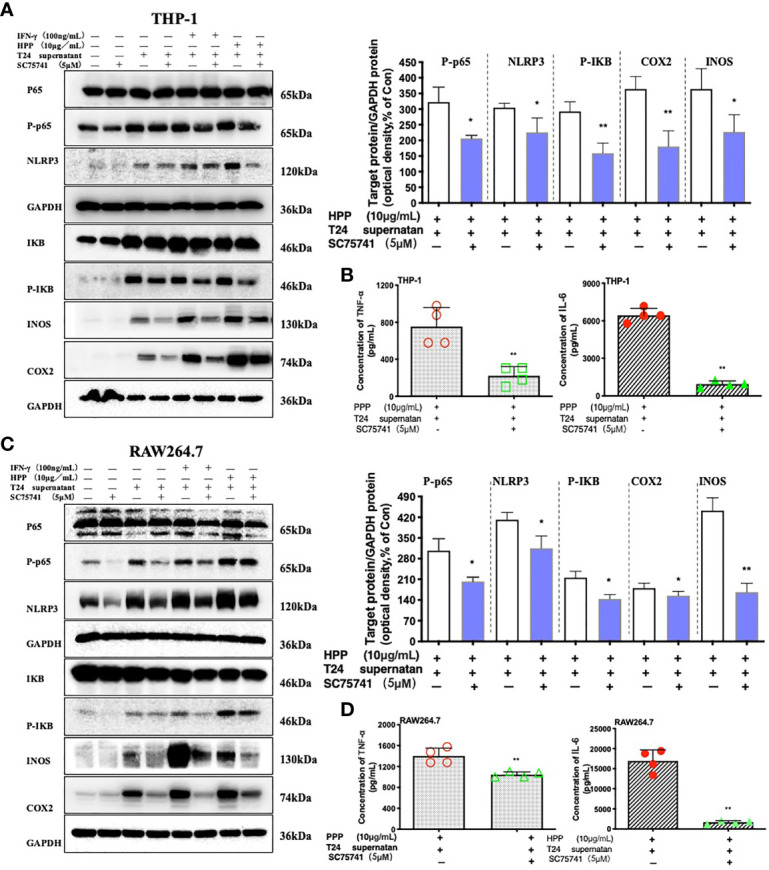
Effect of a specific NF-κB inhibitor on macrophages treated with HPP. **(A, C)** Effect of HPP on NF-κB in RAW 264.7 or THP-1 macrophages. Phospho-P65-NF-κB, NLRP3, phospho-I**κ**B, INOS, and COX-2 were determined using western blotting. **(B, D)** Inflammatory cytokine secretion in macrophages. Values are given as the mean ± SD from three independent experiments. Compared with the control group, ^#^ *P* < 0.05; compared with the RAW 264.7+T24 control group, *P < 0.05.

To further verified whether HPP induced the polarization of TAMs toward M1 by the NF-κB/NLRP3 pathway in the tumor microenvironment. We added inhibitors of the NF-κB pathway, as shown in **Figures 7A, C**. Compared with the control group (SC75741+HPP), the protein expression levels of IκB, Cox2, iNOS, P65-NF-κB and NLRP3 were significantly different (*P < *0.05). In conclusion, by inhibiting the P65-NF-κB protein, the activation of the TAM NF-κB/NLRP3 pathway was reversed by polyporus polysaccharides. As shown in **Figures 7B, D**, the results of the liquid chip assay showed that the secretion of the inflammatory cytokines IL-6 and TNF-α by macrophages was decreased by inhibiting the NF-κB pathway. These results indicate that HPP regulated the polarization of macrophages toward M1 by the NF-κB/NLRP3 pathway axis in the tumor microenvironment.

## Discussion

Immune repression within the tumor microenvironment has gained increasing relevance in cancer research over the last decades. Accumulating evidence shows that TAMs are a major component of tumor stroma and play an important role in promoting cancer proliferation. Monocytes derived from blood or bone marrow circulate throughout the body and are distributed in various organs and tissues of the body. They differentiate and develop into macrophages, which play an important role in the immune response, inflammatory response and maintenance of internal environment stability of the body (22). M1 subtype is characterized by high expression of CD40, CD16/32 and CD86 phenotypic molecules, high secretion of IL-6, IL-12P70, TNF-α, IL-1β and NO and low secretion of IL-10 and TGF-β (8, 23–27). M1 subtype Macrophages secrete pro-inflammatory factors to promote immune response and enhance the effect of killing tumors (7, 8). While M2 subtype is characterized by high expression of mannose receptor MR, SR, CD163, CD204, etc. and high secretion of cytokines such as IL-4, IL-10, and TGF-β (8, 23–25), which is manifested in the suppression of the immune response, chronic inflammation and tissue repair ability. Factors secreted by M2-type macrophages lead to anti-inflammatory effects, alleviate inflammation, repair injured tissues, promote vascular formation, and provide conditions for tumor immune escape (8, 28). A wide body of evidence has suggested that high M2-like polarized macrophage infiltration correlates with poor clinical outcomes in patients with bladder cancer (29). Herein, we established a rat model of BBN bladder cancer, which is one of the most classical models of bladder cancer. The results showed that, consistent with literature reports, CD163 was highly expressed in bladder tumor tissues, while INOS was weakly expressed in microenvironment. Moreover, *In vitro* studies showed that macrophages co-cultured with tumor supernatant were highly differentiated and highly expressed factors related to M2 subtype. These findings provide the evidence that CD163, CCL2 and IL-10 were highly expressed in bladder cancer-associated macrophages.

The polysaccharide of traditional Chinese medicine can activate immune cells and improve the immune function of the body, and it has attracted the attention of many researchers for its precise effect and minimal toxicity and side effects (30). Polyporus used for diuretic in clinic and its main active component is polysaccharide. Evidence from recent studies showed that PPS can be used to treat tumors and reduce the side effects of combined chemotherapy drugs. Previous study had found that the mortality of the Bacillus Calmette-Guérin (BCG)-treated group was higher than that of the group treated with PPS combined with BCG (15), indicating that PPS can reduce the toxicity of BCG. And we found that PPS could suppress the growth of tumor cells (31) and greatly induce the transformation of M2 subtype macrophages to the M1 subtype *in vitro* (17). PPS injection has a good therapeutic effect on tumors. However, there are adverse reactions, such as arthritis. The complex composition of PPS injection is one of the most important causes of adverse reactions. HPP was first isolated from polyporus total polysaccharide, which was proven to have an α-(1→4)-linked D-galactan backbone. Previous work by the team found that PPS has a clear regulatory effect on the polarization of macrophages (21), but the regulatory mechanism in TME is not clear.

TLRs are widely expressed in macrophages and bind with various pathogenic microbial molecules to activate various immune cells through signal transduction and participate in the physiological and pathological process of regulating the TME.TLR2-like receptor can identify exogenous pathogens by binding to lipopolysaccharide, stimulating the production of antimicrobial peptides and inducing nonspecific immune responses, and it is one of the important targets of polysaccharides. The NF-κB (nuclear factor κB, NF-κB) pathways play a central role in the immune and inflammatory processes that regulate the expression of cytokines (21). Previous studies have shown that PPS binds to TLR2 and activates the downstream NF-κB signaling pathway to produce a large number of pro-inflammatory factors. However, whether HPP promotes the polarization of TAMs toward the M1 phenotype trough NF-κB remains unclear. Activated macrophages can secrete NO, which plays an important role in antitumor cells and microorganisms (20, 32). Here, we demonstrated that HPP dose-dependently upregulated the secretion of pro-inflammatory factors such as IL-6, TNF-α, NO, MIP-1α and MIP-1β as well as NO in macrophages trough NF-κB in TME.

The inflammasome is a large molecular protein complex that recognizes pathogenic microorganisms through pattern receptors, activates caspase-1, cuts pro-IL-1β and pro-IL-18, and generates and secretes cytokines corresponding to the immune response of the body. Currently, NLRP1, NLRP3, NLRP6, NLRP7, NLRP12 and NAIP have been discovered, among which NLRP3 is the most studied and most related to immune relationships (33–35). NLRP3 and pro-caspase-1, pro-IL-1β and pro-IL-18 are so low in their resting state that by ligand binding of toll-mode receptors, the transcription factor NF-κB is delocalized into the nuclei and NLRP3 is initiated (36–39). We further showed that HPP activated the NLRP3 pathway in a dose-dependent manner.Therefore, our results provide the insights into the mechanisms of HPP in TME.

**Figure 8:**
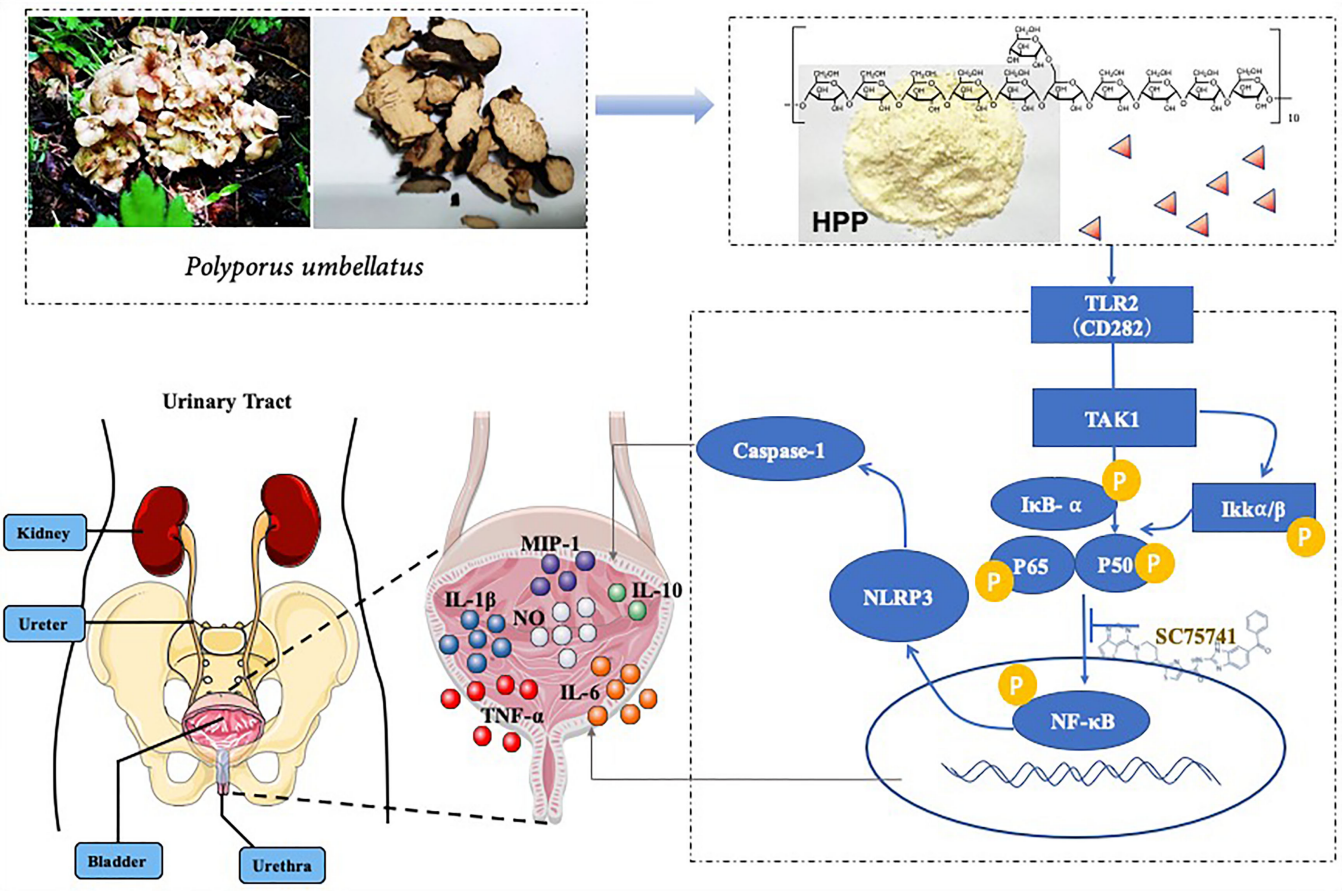
Schematic diagram of HPP promoting the polarization of TAM cells toward M1.

In summary, our study provides evidence supporting that PPS promoted the polarization of TAMs to the M1 subtype and inhibited bladder tumors is shown (**Figure 8**). First, we found that polyporus could improve the effect of BBN bladder cancer rats, which may be related to the induced polarization of TAMs toward M1. Second, a homogenous polysaccharide was extracted and isolated from polyporus. Furthermore, the mechanism of monoclonal polysaccharide was investigated, and it was found that the TLR2/NF-κB/NLRP3 pathway promoted the polarization of TAM cells toward M1 and secreted pro-inflammatory factors to improve the bladder tumor microenvironment. Subsequently, we conducted further verification to determine the mechanism by which HPP promotes TAM polarization toward M1.

## Data Availability Statement

The original contributions presented in the study are included in the article/**Supplementary Material**. Further inquiries can be directed to the corresponding authors.

## Ethics Statement

The studies involving human participants were reviewed and approved by Guangdong Hospital of Traditional Chinese Medicine. The patients/participants provided their written informed consent to participate in this study. The animal study was reviewed and approved by Guangdong Hospital of Traditional Chinese Medicine.

## Author Contributions

CL was responsible for the completion and writing of the experiment. SZ, DH, and HC were responsible for the modification article and data collation.JZ was responsible for the identification of pathological sections. XL and XZ were responsible for the overall design of the experiment. All authors contributed to the article and approved the submitted version.

## Funding

This work was supported by the National Natural Science Foundation of China (No. 81573769), Special project of Guangdong Academy of Traditional Chinese Medicine (YN2019MJ02), Project of Guangzhou University of Chinese Medicine (2021 XK72).

## Conflict of Interest

The authors declare that the research was conducted in the absence of any commercial or financial relationships that could be construed as a potential conflict of interest.

## Publisher’s Note

All claims expressed in this article are solely those of the authors and do not necessarily represent those of their affiliated organizations, or those of the publisher, the editors and the reviewers. Any product that may be evaluated in this article, or claim that may be made by its manufacturer, is not guaranteed or endorsed by the publisher.
