# Supplementary material for: Homogeneous Polyporus Polysaccharide Inhibit Bladder Cancer by Resetting Tumor-Associated Macrophages Toward M1 Through NF-κB/NLRP3 Signaling
Source: Front Immunol. 2022 May 4;13:839460. doi: 10.3389/fimmu.2022.839460 (PMC9115861; doi:10.3389/fimmu.2022.839460)
Supplement: Supplementary file 1 [file Table_1.pdf]

S1.

Table 1. Primer sequences.

Gene	Sense strand (5'-3')	Antisense strand (3'-5')
IL-6	TACTCGGCAAACCTAGTGCG	GTGTCCCAACATTCATATTGTCAGT
INOS	CGGCAAACATGACTTCAGGC	GCACATCAAAGCGGCCATAG
TNF-α	GGGGATTATGGCTCAGGGTC	CGAGGCTCCAGTGAATTCGG
IL-1β	CCATGGAATCCGTGTCTTCCT	GTCTTGGCCAGGACTAAGG
IL-10	TGGGAAGAGAAACCAGGGAGA	GTTTTCAGGGATGAAGCGGC
TGF-β	CCACACCTCTGGGAGACTTC	GTTCCACCAACCCACTGACA
GAPDH	TTTGTCAAGCTCATTTCCTGGTATG	TGGGATAGGGCCTCTCTTGC
